# Online Intergroup Polarization Across Political Fault Lines: An Integrative Review

**DOI:** 10.3389/fpsyg.2021.641215

**Published:** 2021-10-18

**Authors:** Ana-Maria Bliuc, Ayoub Bouguettaya, Kallam D. Felise

**Affiliations:** ^1^University of Dundee, Dundee, United Kingdom; ^2^University of Birmingham, Birmingham, United Kingdom; ^3^Independent Researcher, Dundee, United Kingdom

**Keywords:** issue driven polarization, affective polarization, ideologically opposed camps, collective narrative, intergroup conflict, social identity

## Abstract

We revisit the construct of political polarization and current distinctions between issue-driven and affective polarization. Based on our review of recent research on polarization from psychology, political science, and communication, we propose to treat polarization as a process that integrates the concepts of *social identification* (collective self-definition) with ideologically opposed camps - that is, psychological groups based on support or opposition to specific socio-political issues and policies (related to issue-driven polarization), and that of *ideological and psychological distancing* between groups (related to affective polarization). Furthermore, we discuss the foundations of polarizing groups – and more specifically, the role of conflicting collective narratives about social reality in providing an initial platform for polarization in a technologically networked world. In particular, we highlight the importance of online media in facilitating and enhancing polarization between ideologically opposed camps. As a theoretical contribution, the review provides a more functional conceptualization of polarization that can explain how polarization may occur across partisan fault lines and in domains outside of politics. We conclude with a discussion of new pathways to the study of polarization which this integrative conceptualization opens.

## Introduction

Advances in modern communication technology have enabled wider access and reach across some of the most culturally and geographically remote regions in the world, therefore creating unprecedented opportunities for cooperation that bridges cultural, economic, and geo-political boundaries. However, despite these possibilities, modern societies seem more fragmented and polarized than ever before (McCoy et al., 2018; McCoy and Somer, 2019). This paradox can partly be explained by the anxieties brought on by rapid societal changes, looming economic uncertainty, and existential threats (all significantly compounded by the COVID-19 pandemic). These factors undoubtedly contribute to the highly polarized world we now live in - in particular, by making people more inclined to interpret disruptions, challenges, and conflicts in society through an “us versus them” lens. In the recent literature on polarization, there is a tendency to approach polarization as being either issue-driven (i.e., ideological divisions in society driven by differing policy positions, see Duffy et al., 2019) or affective (manifested as distrust and intense dislike of the outgroup, Levendusky, 2018; Abramowitz and McCoy, 2019; Druckman and Levendusky, 2019). Going beyond this distinction, but building on these current approaches to polarization, we focus on the foundational elements of polarization – those aspects that provide the bases of both issue-driven and affective polarization. By concentrating on the social psychological roots of polarization in the context of a rapidly changing society, we seek to provide an updated conceptualization of polarization that integrates ideas from various disciplinary domains studying polarization and also takes into account the implications of recent technological advances in human communication. To achieve this aim, we review research from social and political psychology, political science, and communication and media studies. Based on our interpretation of this literature, we propose that intergroup polarization can be better understood by addressing three interrelated questions:

(a) When does polarization occur?
(b) Why do particular groups polarize?
(c) How does polarization occur in modern society – given the affordances of rapidly evolving communication technology?

Our review is structured around potential answers to these questions. Specifically, we propose that intergroup polarization is most likely to occur when there is an *ideological conflict* which divides a society. Such conflicts lead to the development of alternative narratives about social reality (often mutually exclusive) which, in turn may provide the bases for group formation. We turn next to the ‘us versus them’ divide and specifically, we discuss how we can best conceptualize the intergroup dynamics that underpin polarization. Specifically, we believe that polarization occurs between groups based on collectively shared support for a particular narrative about social reality (they are driven by intragroup consensus) and which are in opposition to an alternative (conflicting) narrative (they are energized by intergroup dissent). We refer to these groups as ‘*ideologically opposed camps*’ (Bliuc et al., 2020b, 2021), a concept that applies to polarization in the political domain that transcends traditional partisan boundaries (e.g., Brexit) as well as to polarization in other areas of society (e.g., divides on vaccination, animal rights, and climate change). Polarization can be seen as enabled and boosted by social interaction between both people from the same group (intragroup interaction) and people from opposing groups (intergroup interaction). Nowadays, *online social interaction* increasingly determines both the ways in which polarization occurs and its pace. Therefore, we review recent research on the enabling and amplifying role of online communication in polarization in intragroup and intergroup contexts.

## Structure of the Review

We start by revisiting conceptualizations of polarization in social psychology, political science, and other relevant fields, and building on these we propose a working (integrative) definition of polarization that we use in the context of this review. Then, we review recent research on polarization between groups in the context of socio-political issues which are relevant to large segments of population. We first discuss key research that distinguishes between issue-driven and affective polarization; this section is followed by a discussion of emerging research that treats polarization as a process that is both issue-driven and affective, in groups that are ideologically opposed. We include a discussion about the basis of ideologically opposed camps and the role of collective narratives about social reality as providing the foundations of these groups, and (implicitly) the bases for polarization to occur. We complete the review by discussing the role of online communication in facilitating and boosting polarization between ideologically opposed camps. We conclude by identifying directions for future research derived from the new integrative treatment of polarization that we propose.

## Defining Polarization

In social psychology, polarization is traditionally conceptualized as an intra-group process involving deliberation between group members which results in a shift in the group position to become more extreme – that is, after discussions, the positions of group members become more extreme (Moscovici and Zavalloni, 1969). In the social identity approach, and particularly, self-categorization theory (SCT, Turner et al., 1987), the intergroup context is introduced as an essential dimension of polarization. That is, SCT explains this type of polarization through conformity to a *group norm* that is inferred and accentuated through reference to relevant outgroups (Turner et al., 1989; Hogg et al., 1990; see also the concept of referent informational influence, Turner et al., 1987; Abrams et al., 1990). In more recent developments of the social identity approach such as the interactive model of social identity formation (Postmes et al., 2005a,b), group norms develop through both inductive and deductive pathways: a social identity with its associated normative content can be *induced* through communication between ingroup members, but at the same time, it can be *deduced* from the broader context and in relation to relevant outgroup(s). Within the group, polarization may occur through intragroup interaction which in turn leads to ‘consensualisation’ (i.e., enhanced ingroup stereotype consensus); however, even within the group, polarization occurs in a broader intergroup context where the outgroup is salient (Haslam et al., 1998).

Building on these ideas, we propose that consensus about *collective narratives* (shared cognitions and perspectives about the social reality) may provide a platform for inductive identity formation and development. At the same time, intergroup dissent - occurring when ingroup and outgroup hold conflicting collective narratives – may provide conditions for deductive identity content development. We argue that polarization is most likely to occur in groups which are based on consensus about a particular collective narrative about social reality and are defined in opposition to an alternative collective narrative, that is in *ideologically opposed camps* (Bliuc et al., 2020b; see also, Bliuc et al., 2007; McGarty et al., 2009). In the context of this paper, we adopt a broad definition of ideology as “a set of beliefs about the proper order of society and how it can be achieved” (Erikson and Tedin, 2003; p. 64).

In such groups, where exclusive identities are constructed (Goodwin, 2004; Della Porta, 2018), commitment to group position can be represented on an ideological bi-modal continuum, with extreme positions clustering at the end of this continuum – as for example in the cases of polarization between climate change deniers and climate change believers (McCright and Dunlap, 2011; Bliuc et al., 2015; Häussler, 2018), and between supporters of Leave and Remain campaigns in the context of Brexit (Goodwin and Heath, 2016; Hobolt, 2016; Hobolt et al., 2020).

A key merit of the social identity approach to polarization is that it recognizes the roles of both intragroup and intergroup contexts – that is, polarization is a process that occurs within the group, but is enabled and enhanced by intergroup interaction. However, more recent definitions (in particular, from political science) tend to focus primarily on the intergroup dimensions of polarization, as for instance in conceptualizations of political polarization as “both the expanded ideological gap between political groups and the increased interpersonal separation between supporters of opposite parties” (Harel et al., 2020). When strictly applied to partisan polarization (in particular, in the political science literature), it may refer to shifts in *individual* positions, but still in strictly intergroup terms, in the sense of liberals becoming more liberal, and conservatives becoming more conservative (Levendusky, 2009), in a bi-polar fashion. In the social identity approach, this bi-polarity is at least partially explained through a principle known as comparative fit (Turner, 1985; Haslam et al., 1999), according to which groups (and their content) are defined by their extent of similarity/dissimilarity to other groups. That is, according to the intergroup differentiation principle, people self-categorize into groups based on how different they are and are naturally drawn to groups that have the *most* differences between them in that context (Haslam et al., 1999), a tendency that lends itself well to agent-based modeling tests (e.g., Mäs and Flache, 2013). For example, in a gathering of Americans, people could choose to self-categorize into the difference on gender, race, food preferences, or political beliefs, but they will likely choose the group which has the largest difference in that context within that gathering. In that example, a person in this gathering may choose political belief as their metric for self-categorization into a group, as the difference between political beliefs is far larger psychological than the difference between food preferences. This principle also suggests that once self-categorized, individuals (and groups) do what they can to create greater distinctions between their group and other groups (Jetten et al., 1998; Ellemers et al., 1999), thereby defining the group by what separates it from the “outgroup” in the process. Therefore, from this perspective, polarization applied to the political domain almost always occurs in a bi-polar fashion because it is a function of groups trying to distinguish themselves as much as possible from the outgroup (i.e., intergroup differentiation).

In line with the social identity approach, we see polarization as extremitisation of ingroup position (occurring through intragroup interaction and consensualisation) providing an *intragroup consensus driven pathway* (mostly inductive). However, integrating conceptualizations of polarization predominantly from the political domain, we also see polarization as increased distancing from the outgroup (represented by an ideologically opposed camp). These conceptualizations suggest that (intergroup) polarization can result from increasing intergroup differentiation, via a *intergroup dissent driven pathway* (mostly deductive) *–* where polarizing norms are likely deduced from the intergroup context. From this perspective, polarization is a process underpinned by identification with opposed camps in contexts where alternative collective narratives about social reality exists; it manifests in both ideological and psychological outcomes as distancing between ingroup and outgroup in terms of group beliefs, norms, and affective responses (as the social identity content of opposed camps shifts toward stronger ingroup stereotype and further away from the outgroup’s).

The political science literature on polarization distinguishes between affective polarization and issue-based polarization (or polarization driven by policy preferences, see Iyengar et al., 2012; Garrett et al., 2014; Iyengar and Westwood, 2015). Conceptually, the construct of affective polarization is derived from social identity theory (Tajfel and Turner, 1979; Tajfel, 1982) and its ingroup-outgroup distinctions; applied to the United States partisan politics, refers to the tendency of Democrats and Republicans to dislike and distrust each other when political identities are salient (Druckman and Levendusky, 2019; Zoizner et al., 2020). It incorporates the idea of ‘principled dislike toward the outgroup’ (Iyengar et al., 2012). Outgroup hate is not an inherent outcome of group identification (‘ingroup love’); there are many contexts where ingroup favoritism exists, but it is not systematically correlated to outgroup hostility (Brewer, 1999; see also Kosterman and Feshbach, 1989; Hinkle and Brown, 1990). However, treating polarization as a negative outcome of group identification makes theoretical sense if we consider that (ideological) intergroup conflict forms the bases of social identities prone to polarization as well as the existence of both perceived realistic threat (competition over political power) and symbolic threat (competition over beliefs and norms) (Stephan and Stephan, 1996, 2001).

Issue-based polarization on the other hand refers to the bi-modal clustering of positions on important policy or social issues (Duffy et al., 2019). However, by treating polarization as a process of ideological and psychological distancing between ideologically opposed camps, we can *conceptually integrate* the constructs of issue-driven and affective polarization. That is, ideologically opposed camps are psychological groups which are issue-driven (when ingroups and outgroups are shaped by citizens’ issue positions as in issue driven polarization). However, as psychological groups, they entail the intergroup dynamics of ingroup favoritism, and increased antipathy and hostility toward the outgroup (as in affective polarization). In other words, in these groups, polarization can be seen as issue-driven and manifesting as affective polarization (leading to outcomes described as affective polarization, including intense distrust and hostility toward the opposing group).

Recent conceptual developments aiming to explain political polarization primarily in the context of the United States politics, introduce the construct of ‘political sectarianism’ defined as “the tendency to adopt a moralized identification with one political group and against another” (Finkel et al., 2020, p. 533). The basis of this type of identification is similarity in terms of “(…) faith in the moral correctness and superiority of one’s sect” (p. 533). While this construct was developed in relation to political partisanship, a similar process of moralized identification can occur beyond political parties – in particular, in groups which are based on shared collective narratives about social reality (or opposing ideological camps). For example, the dynamics between non-politically affiliated groups such as pro-life and pro-choice can be seen as driven to a large extent by beliefs of moral superiority of one group over the other.

To illustrate this phenomenon and the asymmetry of political polarization in the specific United States context, it may be helpful to consider a particularly salient example: the rise of Donald Trump, a particularly polarizing figure. Across multiple issues, including race and climate change, Republicans and Democrats were fairly similar in 2004, but in 2014, the gap became much wider (during the late Obama years; Pew Research Center, 2014). During these years, the Republican party became more and more polarized, with a clear division occurring within the party- to the point where a new, more conservative camp (the Tea Party) emerged (Arceneaux and Nicholson, 2012), eventually becoming the “voice” of the Republican party (Blum, 2020). This camp defined itself through intergroup interaction and intragroup interaction (obstructing Democrats wherever possible instead of seeking compromise and talking about “draining the swamp” of those who did not abide by their more conservative values). These obstructionist tactics resulted in further hostility and distrust between Republicans, Democrats, and less conservative Republicans, which Donald Trump capitalized on in his rise to power (Blum, 2020). Donald Trump also capitalized on and engineered greater polarization by being as divisive as possible within his own party; his main message that America had lost its way because of attacks that reduced Americans’ statuses resonated with voters. In fact, status threat-fear that “we” (as Americans) were losing “our way” was the single greatest contributing factor in predicting votes for Donald Trump over everything else (Mutz, 2018). This example suggests that this outcome of polarization as a sequence of events following ideological conflict through division in camps does have descriptive power, and we should consider how issue and affective polarization play out together and separately.

Finally, research from communication and media studies provides evidence that modern communication technologies act as a catalyst for polarization primarily by facilitating intense intragroup interaction – thus, validation and extremization of group position in ‘echo-chambers (Wojcieszak, 2010, 2021; Wojcieszak and Garrett, 2018), but also by making intergroup conflict more salient through exposure to outgroup’s views (Han and Federico, 2017; Wojcieszak et al., 2018, 2021). We discuss in detail the evidence for both these pathways to polarization later in the article.

**FIGURE 1:**
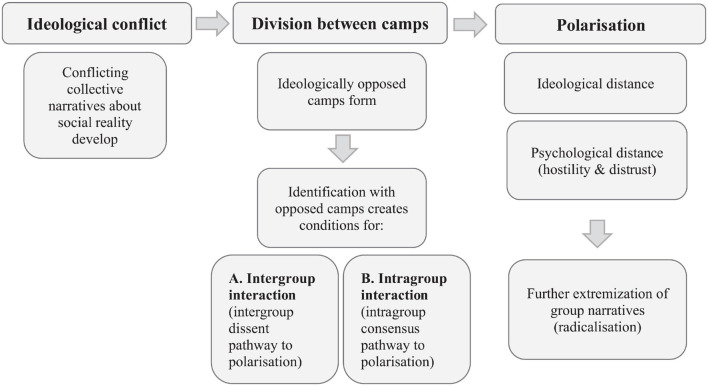
Polarization, represented as a process initiated by the existence of conflicting narratives about social reality (ideological conflict), underpinned by division between members of opposed camps, and manifested as ideological and psychological distancing from the outgroup. Radicalization could occur as a result of further extremization of group narratives.

In summary, as illustrated in Figure 1, polarization may be represented as a continuous process that starts with dissent between ideologically opposed camps when conflicting collective narratives about social reality develop (ideological intergroup conflict). As individuals start to identify with these conflicting narratives within ideologically opposed camps, division between these groups occurs and is accentuated by intragroup processes of validation and consensualisation (including in the online domain in ‘echo-chambers’) and by intergroup processes of exposure to the opposing position of the outgroup; polarization in this case manifests as increasing psychological and ideological distance (through increasing of outgroup stereotyping and discrimination leading to outgroup hate and distrust, and respectively, ideological positions becoming harder to reconcile). Over time, growing ideological distancing can also lead to radicalization of group beliefs – i.e., the group position becoming increasingly extreme – in particular, when the ideological conflict and subsequent polarization occurs between people in the same group, as shown in research on intragroup conflict and polarization (Groenendyk et al., 2020). However, while Figure 1 illustrates a theoretical pathway to polarization, it does not mean that ideological conflict in society and identification with opposed ideological camps is always resulting in polarization and possibly radicalization – that is, there are alternative pathways that can lead to unification and de-radicalization, as for example when contextual changes create conditions for identification with superordinate categories, for the need to achieve common goals (e.g., climate change deniers and believers can unify to achieve common goals such as clear water provisions, see Postmes, 2015) or when a shared threat arises (Brewer, 1999).

## Research on Polarization as Either Issue-Driven or Affective

Issue-driven polarization (also known as ideological polarization, Webster and Abramowitz, 2017) refers to the divisions formed around policy positions or issues which are important in society to a large section of the population (Duffy et al., 2019); it is bi-modal, in the sense that it occurs around two distinct positions, and support is clustered around either of these positions. Increasingly, in the context of United States politics, it has become apparent that polarization conceptualized (and measured) as voters’ position does not fully capture current partisan conflicts (Druckman and Levendusky, 2019). The construct of affective polarization represents an extension of the idea that polarization is the process whereby liberal and conservative positions are divided across support and opposition to various policy issues and positions toward these issues, and it is reflected in the clustering of these positions toward the opposite ends of the political spectrum. More than support and opposition to these issues, polarization manifests in ‘affective terms’ as intense dislike of the outgroup; it implies “sympathy to the in-party and antipathy toward the out-party” (Abramowitz and McCoy, 2019, p. 2, see also Huddy and Yair, 2021). In particular, affective polarization “requires not only positive sentiment for one’s own group, but also negative sentiment toward those identifying with opposing groups” (Iyengar et al., 2012, p. 819; see also, Klar et al., 2018). This highlights a novel development in modern politics – partisan attitudes and behaviors might be driven more by dislike of the outgroup rather than affinity for the ingroup (i.e., the rise of negative partisanship reflected in ‘Us versus Them’ divisions of the public, Abramowitz and McCoy, 2019). As noted by Iyengar et al. (2019):

Ordinary Americans increasingly dislike and distrust those from the other party. Democrats and Republicans both say that the other party’s members are hypocritical, selfish, and closed-minded, and they are unwilling to socialize across party lines. This phenomenon of animosity between the parties is known as affective polarization (Iyengar et al., 2019, p. 129).

However, research on affective polarization has found that while voters from the other party might be disliked to various degrees, it is the political elites that are the most strongly disliked and distrusted (Levendusky, 2018; Druckman and Levendusky, 2019). As way to measure affective polarization, several indicators are generally used: (i) *affective ratings* – captured using survey tools such as the ‘feeling thermometer rating’ (asking study participants to rate how cold or warm they feel toward the Democratic Party and the Republican Party, see Lelkes and Westwood, 2017); (ii) *trait ratings* (of how well various traits describe the parties) with positive traits including patriotism, intelligence, honesty, open-mindedness, and generosity, and negative traits including hypocrisy, selfishness, and meanness (Iyengar et al., 2012; Garrett et al., 2014); and (iii) *party trust ratings* (rating the extent to which participants trust the parties to do what is right, Levendusky, 2013). Affective polarization also incorporates the classic concept of ‘social distance’ introduced by Bogardus in 1947 (Iyengar et al., 2012) which captured in modern polarization measures by using questions about how comfortable people are living in close proximity to members of the outgroup (e.g., how comfortable people are in having close friends from the other party, having neighbors from the other party, and having their children marry someone from the other party, see Iyengar et al., 2012; Levendusky and Malhotra, 2016). Furthermore, recent research on affective polarization in the context of the current COVID-19 pandemic (Druckman et al., 2020, 2021), shows that partisan animosity is predictive of both attitudes toward the pandemic (i.e., participants’ concerns about the virus, and support for policies aiming to limit the spread of infection) and behaviors taken to limit the spread of infection, suggesting that at least in the context of United States politics, partisan animosity and issue-positions seem to be aligned and polarized across the expected fault-lines.

### Summary

Issue-driven polarization is primarily defined as divisions formed around policy positions or issues that matter to social groups within a society, with bimodal clustering around opposing poles. However, this view is limited as research on issue-driven polarization has shown that position alone does not fully encompass current partisanship behavior and attitudes. Meanwhile, affective polarization is largely the extent of intense dislike for the opposing group, with a much more positive view of the ingroup. The dislike is especially powerful, as it appears more recent research suggests that negative affective polarization (driven by dislike of the other group) might be more predictive of partisan attitudes and behaviors than like of one’s own group. Both these streams of research have shown significant shifts in how polarization is developing and changing and considering a new theoretical approach may be more descriptive and predictive of polarization trends.

## Polarization Between Ideologically Opposed Camps

Scholars agree that there is increasing polarization in American politics and beyond, but they are still debating about the ways in which polarization can be best captured: by focusing on relations between attitudes (as in issue polarization) or by focusing on relations between people from opposing parties (as in affective polarization) (Mason, 2016). There is recent evidence that issue-driven and affective polarization may be connected. In particular, it seems that attitudes toward policy issues (such as social welfare, for example) have become increasingly aligned with partisan identification (Webster and Abramowitz, 2017). Similarly, attitudes on key issues in society such as climate change seem to be divided across partisan lines and increasingly consistent with partisan ideologies (McCright and Dunlap, 2011; Dunlap et al., 2016). As partisan identities are evolving to become increasingly aligned to other social identities (religious, racial, ethnic, etc.), a process of convergence or ‘social sorting’ takes place/unfolds (Mason, 2015, 2016; Davis and Dunaway, 2016). That is, social sorting occurs “when religious, racial, and other political movement identities grow increasingly linked to one party or the other (…)” (Mason, 2016, p. 351). For example, non-Hispanic white people and born-again Christians in the United States make up a much larger share of Republicans than Democrats (Egan, 2020). Not surprisingly, there is an emerging trend in the field to treat polarization as interconnected stages of the same process, rather than distinct constructs that capture different processes (Rogowski and Sutherland, 2016). For example, in recognizing the importance of ideology in the process of polarization, Lelkes (2021) notes: “(…) elite polarization and partisan sorting have driven Americans to see large policy differences between their side and the other side (…), making affective polarization a logical outcome.”

### The Role of Collective Narratives in Polarization

However, going beyond the ideas of interconnectedness and continuity between issue-driven and affective polarization, a comprehensive conceptualization of polarization must include instances of polarization between groups defined by support for issues that cut across political fault-lines. One example of such groups is the anti-vaccination movement (Schmidt et al., 2018) which includes both conservatives, who may be primarily driven by religious values, and liberals, who may be primarily driven by libertarian values (Rossen et al., 2019; McCoy, 2020). Another example of polarization which is not fully captured by partisan divides is the social movement for Scottish independence, where for the 2014 referendum, supporters for the independence (‘Yes’ movement supporters) were not only drawn from the Scottish National Party (SNP), but also from other parties such as the Liberal Democrats, Labor, and even Conservatives (Dalzell, 2018).

While these movements are clearly issue-driven and likely energized by ideological opposition (as in issue driven polarization) and hostility toward the outgroup (as in affective polarization), neither of these theoretical approaches can fully explain polarization between anti-vaxxers and their opponents. Nor can these approaches fully explain polarization between ‘Yes’ supporters and their opponents (‘unionists’). A theoretical explanation that accounts for issue-driven polarization that cuts across political partisan boundaries must start with the root of the issue; thus, to understand polarization, we need to identify the *bases of groups that polarize* – that is, the bases of ideologically opposed camps. We propose that these bases are endorsement of specific *collective narratives* about social reality. These narratives are collective in the sense that they are shared between like-minded others and collectively endorsed, and as such they may provide bases for social identity formation (Bliuc et al., 2020b) or unification of disparate but compatible identities under (as for example, in the case of Brexit supporters who were drawn from various pre-existing euro-skeptic groups). Such narratives are central to understanding the psychology of polarization, because they can help us understand the psychological processes that drive individuals to identify with a collective cause and behave in line with social norms that are prescribed by particular groups (as per social identity theory, Tajfel and Turner, 1979). Importantly, they provide individuals with meaning and structure through rich, but cohesive stories that help them interpret social reality in ways aligned to their moral values, pre-existing beliefs about society (and the world more generally), and social identities based on social categories (such as class, gender, profession, etc.). Present and past social reality can always be explained or interpreted through more than a single narrative, and in most cases the available narratives include conflicting arguments that often make these narratives *mutually exclusive*.

A clear illustration of the power of alternative and mutually exclusive narratives to polarize the public is provided by the 2020 United States presidential elections. As the elections were unfolding in November 2020, there were currently two dominant narratives, both equally legitimate in the eyes of their respective supporters, describing alternative realities that, in this case, were mutually exclusive: in one version of reality the elections were democratic and procedurally fair, resulting in Joe Biden indisputably winning the elections, while the other version presented a completely different reality where the elections were compromised by unlawful interference and corruption, with Joe Biden’s win being strongly contested. Similarly, climate change deniers identify themselves as skeptics (Bliuc et al., 2015; Fielding and Hornsey, 2016) and have strong group identities which are based on a particular narrative about a social reality where, for instance, global warming is not caused by human action. Climate change deniers define their identity in an oppositional manner to ‘climate change believers’ and they are driven in their behaviors by emotions, moral values, and norms which are aligned to their group identity (Bliuc et al., 2015).

Within a person, any number of collective narratives may converge and together contribute to a personal narrative that makes people who they are as unique individuals. A personal narrative would include elements which are unique to the individual (e.g., perceptions of personal traits and experiences), but also collective narratives with varying degrees of centrality to self-definition that contribute to self-concept – that is, the self-concept can be seen as incorporating many roles that are represented as social categories (Markus, 1977; Turner, 1985; Turner and Reynolds, 2010) – e.g., as a parent, party member, citizen of a particular country, etc., but also endorsement of specific narratives that provide information about what a person is ready to stand for relative to other people in society (McGarty et al., 2009). Returning to the context of polarization, collective narratives are particularly important because they embed specific (collectively shared) moral values, beliefs, and norms that shape attitudes toward the outgroup and guide individual behaviors and collective actions. That is, collective narratives inform the content (values, norms, and behaviors) of the respective social identities that develop from support of these narratives. A recent illustration of research on polarization between ideologically opposed camps that also conceptually integrates issue-driven and affective polarization is provided by a study of polarization around Brexit in the United Kingdom (Hobolt et al., 2020). In this work, the construct of polarization is extended by investigating polarization between groups based on their position toward Brexit (i.e., supporters of the Leave versus supporters of the Remain campaign), in addition to partisan affective polarization. Data from United Kingdom participants placed at both sides of the divide shows that social identities based on ideological positions toward Brexit were more relevant than partisan identification: Brexit identities were considered personally more important by the participants, and they generated affective polarization as intense as it would be expected from partisan divisions, as indicated by increases in prejudice, stereotyping and other indicators of negative intergroup attitudes.

In some cases, collective narratives may take over social identities based on social categories such as national identity (so that a social identity can be fully expressed as support and enactment of a particular narrative). The best illustration of such cases is when national identities become dominated by collective narratives about identity and the meaning of key historical events are based on interpretations, not shared by the whole nation. For example, research on national identity, shows that in the context of Australia, intergroup conflict driven by contrasting narratives about what it means to be an Australian may divide the community into supporters versus opponents of multiculturalism; these collective narratives provide opposing definitions of the Australian identity and opposing arguments (with associated beliefs, values, and norms) about what social identification as an Australian entails (Bliuc et al., 2012). Similarly, dissent about narratives incorporating the meaning of historical events currently celebrated during the Australian national day (and the legitimacy of this celebration) divides the Australian community into supporters and opponents of “Change the date” – a campaign supporting changing the current date for celebrating Australia day from January 26, a date signifying the arrival of British colonizers and the beginning of modern Australia (but at the same time the start of a dark historical period from the perspective of the Indigenous population) to a date that could be equally celebrated by Indigenous Australians (Bliuc et al., 2020b, 2021). Similarly, in the context of intractable conflict, alternative narratives about past suffering, guilt, and justice further polarize citizens of nations such as Israel and Rwanda (Rouhana and Bar-Tal, 1998; Bar-Tal et al., 2014; Moss, 2014).

However, because collective narratives can evolve and change, the social identity content of ideologically opposed camps is also fluid implying that polarization between different camps and outgroup hate are not universal outcomes. Narratives about national identity for instance can change to incorporate unifying values and norms and therefore providing a platform for camps to expand and unify. We have seen this recently in relation to England’s football team in the EURO 2020 through the deliberate messages of anti-racism, promoting an inclusive, unifying version of what it means to be English (Smith, 2021). Similarly, instances of unifying collective narratives are seen in the Black Lives Matters and MeToo movements which bring together people from different social categories (across ethnic boundaries and respectively gender categories). Therefore, a collective narrative approach to polarization incorporates potential solutions to decrease or even reverse polarization – it is only when there are no alternative unifying narratives available that polarization between camps represents the unavoidable outcome.

### Summary

A collective narrative approach to polarization appears to have several advantages over accounts that distinguish between issue-driven and affective polarization. This approach suggests that by considering the social psychology of collective narratives, we can better understand the bases of relevant social identities, which then links to and bridges the affective and issue-driven approaches to polarization. The collective narrative around issues within a social identity can drive group formation and polarization between groups as one’s social identity based on one’s ideological position may be the root of the process. Furthermore, collective narratives contain values, beliefs, and norms that affect intergroup attitudes (they provide the *why* polarization may occur). This collective narrative approach can, therefore, predict group formation and intragroup dynamics on the basis of issues that ingroup members agree in (intragroup consensus), and predict intergroup behavior by evaluating the content of the values and norms embedded within these collective narratives in these social groups (driven by intergroup dissent).

## The Role of Online Media in the Polarization of Ideologically Opposed Camps

Collective narratives about various socio-political issues or aspects of social reality can emerge through any medium where human interaction is possible, but polarization appears to be accelerated through online interaction. Communication via online media has recently become a more potent method of polarization with much wider effects than other forms of media and understanding how online media is different to other modes of communication may aid in explaining increased rates of polarization in recent years. Online environments may affect the likelihood of polarization through their influence on the pace and quality of the transformation of collective narratives in ideologically opposed camps (when they are communicating online). Recent theoretical developments from communication and media studies suggest that transformative political behavior might be undermined by the ‘chaotic pluralism’ driving a myriad of online micro-actions (‘tiny acts’ of political participation) such as sharing, liking, donating small amounts for various causes, and so, which while they create turbulence, their impact on decision-makers may be limited (Margetts, 2016; Margetts et al., 2016). These behaviors are seen to be underpinned by complex non-linear dynamics and randomness, so are likely harder to model and predict; however, even in these highly complex (online) environments, we know from research based on the social identity of deindividuation effects (SIDE, Reicher et al., 1995; see also Klein et al., 2007) that even small behaviors in these contexts are still driven by salient social identities. Thus, in a sense, our approach to understand the ‘us versus them’ dynamics of polarization as driven by competing, ideologically driven social identities can offer solutions to modeling and predicting the potential impact of seemingly disparate political micro-actions online.

In online media, people can engage with a wider range both like-minded others and people who support opposing narratives than ‘in real life,’ while outside of the digital world, people spend time within relatively homogenous groups, marrying and befriending people within these very same groups (Kalmijn and Flap, 2001; Alves et al., 2016; Luo, 2017). By contrast to the offline contexts, online media provides a rich platform for the development of and refinement of various collective narratives online. By facilitating social interaction, and therefore, the processes of deliberation, mutual validation, consensualisation, and endorsement of arguments and overall narratives, these processes contribute to add nuance and complexity to narratives.

There are three key factors that enable this in the online world. First, it is easier to communicate and connect with like-minded others online, as often (ideologically driven) virtual communities are brought together by consensus or support for a particular narrative about social reality over others. Unfortunately, this often acts as a method of validating fringe beliefs through social proof, but it also provides the proverbial glue that holds these groups together (Code and Zaparyniuk, 2010; Eddington, 2018; Hodge and Hallgrimsdottir, 2020). Second, online public spaces have low barriers for entry with a much larger reach, whereas offline public spaces have higher barriers for reaching a larger number of people (usually through established methods of mail, loudspeakers, events, etc.). Thirdly, near anonymity allows and in the case of some online platforms, encourages (e.g., sites/apps like Whisper; see Asenbaum, 2018; Mondal et al., 2020) – people to voice opinions that may otherwise invite social drawbacks in offline settings. Together, these factors suggest online environments are unique in their characteristics, and these characteristics may affect polarization.

When ideologically driven online communities first emerged (especially around hate speech), commentators often dismissed their impact as illusionary groups or spoke about them in abstract terms (Gosnell, 1997), partially because of the limited exposure of the internet amongst these commentators and partially because of the lack of effective internet penetration at the time. However, more recently online media has demonstrated to be far more powerful in polarization than even more recent commentators believed, as the internet has become ubiquitous (especially as a consequence of the current global need for social distancing to prevent COVID-19’s spread), and online polarization has real life consequences. For example, Cambridge Analytica successfully used online media to help polarize opinion of voters from opposing sides toward more extreme views, swaying elections in multiple countries, including India, Pakistan, the Philippines, and Trinidad and Tobago (González et al., 2019). During COVID-19, anti-maskers became a vocal group online, and organized rallies, causing significant public health crises in a number of countries (Jamison et al., 2020; Pleyers, 2020). Black Lives Matter protestors successfully hijacked the use of the hashtag #alllivesmatter and used the counter hashtags of #blacklivesmatter as a way to mobilize support by framing it as a counterprotest (Gallagher et al., 2018). These actions would largely not be possible offline, as the level of organization and reach to mobilize these events would be limited by cost and time.

While much of the research on polarization has focused on western democracies and the increase in polarization between liberal and conservative groups, these patterns of polarization also exist in the rest of the world, driven by the online environment. However, polarization in these countries do not always reflect traditional “liberal” versus “conservative,” suggesting that online polarization does not only magnify this divide, but other differences as well. For example, Malaysia’s increasing polarization caused a collapse of the secular ruling party in March 2020 (Saravanamuttu, 2021). The ruling party Pakatan Harapan (PH) was an inclusive, multi-ethnic collation, but online campaigns resulted in successfully sowing division between and within camps leading the government’s collapse. In Kenya, polarization occurred against ethnic lines, rather than issues; this was stoked by Whatsapp messages shared by the population (Kibet and Ward, 2018). In Turkey, limited evidence suggests that online polarization follows a pattern of support for secularism versus autocratic religiousness (Kutlu, 2021). This pattern of online-driven polarization appears to be similar across the globe, with one analysis suggesting that polarization is much stronger now than at any point in the past 100 years— largely due to the internet (Carothers and O’Donohue, 2019).

Therefore, understanding how online environments can affect polarization is not only critical in the current context, but also provides a powerful illustration of our theoretical argument: in societies divided across the fault-lines of ideological conflict, groups based on consensus about and support for a particular collective narrative over others (ideologically opposed camps) give isolated individuals a platform to form common cause with like-minded others and through communication (online social interaction) endorse and further refine these narratives. As group members, these individuals’ behaviors would be shaped by collective values, beliefs, and norms aligned to these narratives – resulting in increasing polarization as animosity and hostility toward the outgroup, and distancing (ideological and psychological) from the outgroup. As stated earlier, according to our argument, polarization in ideologically opposed groups is both issue-driven and leading to affective polarization. Online groups, which can be categorized as ideologically opposed camps by their nature, are issue-driven in their formation, and we because of the rich digital footprint they leave, provide opportunities to study more comprehensively both intergroup and intra-group polarization by examining the content of these groups over time. Furthermore, we can observe the increased ideological and psychological distancing as these groups interact with opposing others. Using our framework, we review evidence on how online environments can increase the pace and severity of polarization through increasing the likelihood of intragroup and intergroup contact.

### Online Social Interaction With Ideologically Similar Others (Intra-Group) – Effects on Polarization

Online collective narratives help inform social identity content, in the sense that we rely on what others say and do to develop a shared understanding of the world. However, classic social psychological studies refer to group polarization as a process by which group members become more extreme after interacting with fellow group members (Myers and Bishop, 1970; Lamm and Myers, 1978; Isenberg, 1986), and an online environment may serve to accelerate this intra-group polarization process. This effect is likely magnified in an online environment for three reasons: first, it provides opportunities above that of “real life” as there are more opinions and social proof for even marginal opinions, and second the systems (i.e., technology and algorithms) involved with online social media are designed to optimize engagement (which they can do by presenting information that corresponds with or relates to a person’s worldview). Accordingly, the diversity of online ideologically opposed camps tends to be far larger than in real life. Third, early research also suggests that the anonymity (afforded by online social interaction) increases intra-group polarization effects (Spears et al., 1990; Lee, 2007) – a finding later confirmed by research on the effects of echo-chambers in polarization (Flaxman et al., 2016; Quattrociocchi et al., 2016; Del Vicario et al., 2017).

Social media sites may tend to present a wide variety of viewpoints in the direction of polarization for the user (Park and Kaye, 2017) rather than ideologically oppositional content, as the ruling algorithms tend to prioritize presenting content that the user has shown interest in past interactions (Willson, 2014). One particularly large study on American Facebook users (*N* > 10 million) found that Facebook tended not to show much “cross cutting” content (i.e., content that challenged a user’s worldview), partially because of algorithms designed to increase engagement, but mostly because people tend to be friends with those who share similar viewpoints (Bakshy et al., 2015). Similar results were found with Twitter users (Himelboim et al., 2013). This leads to the majority of content on social media—the primary way people socially engage with the internet— fitting one’s perspective of the world. Similar research on YouTube users (*N* > 12 million) found that people online tended to consume content that fit their perspective (Bessi et al., 2016). Furthermore, examination of YouTube’s algorithm suggests that it tends to show more extremist content upon consumption of one extreme video to the point where complete immersion in extremist content is possible by following the suggested videos (O’Callaghan et al., 2015). Therefore, social media may serve to present more viewpoints that would be considered extreme (or polarized) in one direction.

Multiple recent studies suggest, in the context of these algorithms, online interaction with like-minded others increases polarization, possibly more than interacting with others in traditional (i.e., analog) means. On Reddit, membership in some political subsites (known as subreddits) also seems associated with polarization of opinions, as they frequently link to other extreme subreddits [e.g., Reddit’s conservative subreddit often linked to WhiteRights, a white supremacist section (Soliman et al., 2019)]. Another temporal analysis on thousands of abortion tweets suggested that online interaction with likeminded individuals tends to strengthen their group identity and viewpoint— especially as they were exposed to more diverse viewpoints from before (Yardi and Boyd, 2010). Therefore, online environments likely increase intragroup polarization, probably by presenting more diverse views within the ideological boundaries of the group (views that fit one’s perspective).

### Online Social Interaction With Ideologically Opposed Others (Inter-Group) – Effects on Polarization

Inter-group polarization seems similarly accelerated by the online environment, due to the same reasons underpinning the acceleration of intra-group polarization. It has been established that online systems can have an incentive to decrease social interaction with content that opposes one’s initial stance, but they do still allow users to interact with people outside of one’s ideological camp (e.g., via the comment section of a news article or blog). In fact, there is evidence showing that intergroup exchanges of information are more frequent in online media than initially thought (Barberá et al., 2015; Tucker et al., 2018; Barberá, 2020). This may happen because of the increased exposure to information from weak ties (relatives, co-workers, acquaintances, etc.) which are more likely to spread ideologically diverse information (Barberá, 2014, 2020). Theoretically, exposure to content that contradicts one’s worldview would lead to less polarized (moderated) views; well-established research on intergroup contact amongst ideologically intolerant people outside the digital world suggests moderation of their views as an outcome (Hodson, 2011). However, this does not appear to be the case online. Intriguingly, algorithms designed to increase virality have an incentive to provide content that increase anger, as research has demonstrated that online content that makes people angry is far more likely to be spread and shared (Berger and Milkman, 2009). This suggests there is an incentive for systems to both present information that fits one’s initial stance, but also at least some information that counters one’s worldview to increase engagement. A natural consequence of this may be that people online from one ideological stance are exposed more frequently to cross-cutting stories and comments that make people angry due to contrast with their ideological stance; here, we review research on how intergroup polarization is affected by the online environment.

While direct contact with ideologically opposed others in real life can serve to reduce (Hodson, 2011) or increase polarization (Paluck, 2010), as stated earlier, the research into online interaction suggests a starker view: online interactions with others seem to largely galvanize more extreme views, possibly due to a stronger awareness of political or ideologically driven identities in social media interactions (Settle, 2018). For example, one Twitter based study showed that during the 2016 presidential debates, polarization increased dramatically afterward as a result of exposure to intergroup contact (Primario et al., 2017). Another Twitter-based experiment found that Republicans (*N* = 751) expressed more conservative views after being paid to follow a left-leaning Twitter bot compared to a control group, presenting experimental evidence that exposure to intergroup views increases polarization (Bail et al., 2018). Similarly, direct dissent with a comment on a YouTube video relating to changing the date on Australia day in previously mentioned research (Bliuc et al., 2020b) drove polarization. These studies suggest that viewing information that contrasts with one’s world view increased polarization as a form of intergroup contact.

There is a possibility that some groups become more polarized through intergroup contact, while others tend to become more polarized through intragroup contact, or in some cases, both. In an earlier cited experimental study (Bail et al., 2018) paying participants to follow a contrarian twitter bot, the researchers attempted to perform the same experiment with an otherwise similar sample of Democrats (*N* = 908), but found it was insignificant in predicting polarization. In a Facebook based Brexit study (*N* > 1.5 million), the main driver for polarization for Remainers appeared to be echo-chambers, where there was little cross cutting. Meanwhile, Brexiters tended to frequently engage with ideological adversaries (Bossetta et al., 2018). The difference here may be due to the difference in collective narratives and therefore, social identity content; in some groups, being adversarial may be part of the norm, while others may focus more on intragroup cohesion.

### Summary

Overall, the argument that polarization is amplified by online conditions is empirically supported by research. Online forums and sites increase intragroup polarization; views within groups become more extreme as sites tend to provide more opportunities with engagement with like-minded others while also presenting information popular within that community (which only leads to more polarization). Meanwhile, online environments also increase the chances of intergroup polarization because of the diversity and strength of the opinions being expressed. Therefore, online environments increase polarization through social identity and group-based processes, mostly by scaling up opportunities for intragroup polarization and enhancing intergroup polarization through the medium’s own parameters.

## Discussion

Democratic societies have a lot to lose because of the growing polarization. While diversity in opinions and ideologies is a sign of a healthy democracy, increasing polarization about issues that are important for the society and individual (Liu and Latané, 1998) is concerning. In particular, at the level of society, polarization manifests as escalating intergroup conflict that in turns results in increasing fragmentation (i.e., social and psychological distancing between members of ideologically opposing camps). At an interpersonal level, polarization translates into increased antipathy and hostility toward members of the outgroup. While these direct effects of polarization are well documented in the literature on issue-driven and affective polarization, there are potential indirect impacts on society: for instance, increased fragmentation of nations and communities provides a clear pathway to alienation (including self-alienation) of subgroups and therefore provides potential pathways to radicalization (Blackwood et al., 2012; Bélanger et al., 2019). That is, polarization can be understood as an initial step in the process of extremization of collective narratives, which in turn can transform groups into platforms for radicalization to political violence (Smith et al., 2020). Even when polarization does not lead to such drastic effects, it does have long-term ramifications affecting the ability of communities to work cooperatively toward superordinate goals that would not only be beneficial for specific groups or even nations, but for the humanity as a whole (e.g., eradicating child poverty, achieving generalized social justice, and reducing carbon emissions).

In this review, we integrate conceptualizations of issue-driven polarization (focusing on the roots of polarization) with conceptualizations of affective polarization (focusing on intergroup and interpersonal manifestations of polarization), by proposing that polarization can be understood as a process of increased animosity and ideological and psychological distancing between ideologically opposed camps. We highlight the role of ideologically opposed camps – that is, psychological groups formed around support for a particular collective narrative about social reality (Bliuc et al., 2020b, 2021) – in the process of polarization. The interplay between collective consensus and dissent is key in understanding how ideologically opposed camps form: it is consensus that brings like-minded people together to share a collective social identity (identify as supporters of a particular ideological camp), but it is dissent with an opposing side that makes group formation possible in the first place and provided the group with a common cause (something to fight for), therefore keeping the group together and maintaining the intergroup conflict. The collective narratives that underpin these groups are embedded with values, beliefs, and norms, and their transformation (through social interaction and communication) can help us better understand and even predict changes in the actions of polarized group members.

Our re-conceptualization of polarization is placed in the contemporary context where social interaction is dominated by online communication technologies. We show how the process of polarization is amplified by online media through enabling ready accessibility and wider reach to communication with both ingroup and outgroup members. Increasingly, studies show that online communication both with like-minded others (members of the same camp) and with supporters of oppositional views (members of the ideologically opposed camp) leads to heighten polarization between groups. This trend is particularly worrying when the polarizing groups include already extreme communities such as far-right and neo-Nazi forums (Wojcieszak, 2010; O’Callaghan et al., 2015; Bliuc et al., 2019, 2020a) where further polarization and radicalization can result in acts of political violence being committed against members of cultural or religious minorities (as exemplified by the 2019 mosque attacks in Christchurch, New Zealand, where the perpetrator was at least partly radicalized online).

### Future Directions

The proposed theoretical approach on polarization provides new avenues for research. By consolidating research from political science, psychology, and communication, future research can establish whether we can predict when polarization is likely to occur both online and offline, and because recent advances on the use of online tools for academic research (and access to enormous amounts of online social interaction data – including on historical interactions), we can track polarization in real time against this framework.

To refine our current understanding of the link between online polarization and offline events, further research should consider using Twitter hashtags in response to a global event and examine a network of tweets against a social identity map (Bentley et al., 2020). This will serve as a test of hypotheses derived from our conceptualization of polarization as stemming from dissent on alternative collective narratives. In particular, the formation of identities online can be studied as well as the effects of identification with ideologically opposed camps. Future studies can establish whether the formation of a social identity organically arises from issue-based perspectives, and whether the content of the narrative predicts emotional responses from opposing camps. Because this research also uses social psychology research to understand polarization, it can also use social psychological research methods to test predictions on what would work to reduce polarization. There is some early experimental (online) evidence that suggests that making non-issue driven identities salient (such as shared identities as Americans) may reduce partisanship (Levendusky, 2018). The social identity approach also suggests that recategorization has utility in reducing perceived intergroup differences; for example, asking individuals to think of themselves as a member of multiple groups reduces salient differences, potentially reducing more extreme outgroup beliefs (Prati et al., 2016). Therefore, getting social groups to refocus on different collective narratives embedded in different social identities may serve to reduce polarization as well.

Another area of research that requires further examination is the different patterns of polarization in different groups. As stated earlier, research on Brexit online found that Remainers and Leavers differed in their intergroup and intragroup behaviors (Bossetta et al., 2018), while Democrats were less likely to show polarization than Republicans in response to a contrarian Twitter bot (Bail et al., 2018). Together, these suggest that some collective identities may have been energized by different narratives that then lead to different patterns of polarization. We observe that more right-wing groups tended to engage with intergroup members and were more likely to be polarized, suggesting that there may be a narrative around *being* oppositional or reaching out that drives greater polarization in some groups and not others, while other groups may have a narrative around “looking within” to cause more polarization. Alternatively, it may be that some groups on one pole tend to attract certain types of individuals who believe in narratives around outreach (framing themselves as opposition), while others believe in narratives around intra-group cohesiveness (framing themselves from within group discussion). The difference between these hypotheses is that the former hypothesis is around the narrative that drives member behavior, while the latter suggests that the members attracted to certain narratives tend to behave in a different way. Measuring these values and member behavior before and after being part of a group may provide clarity on how collective narratives link to polarization, and therefore, can act to refine the model presented in this paper.

## Conclusion

In this review, we present evidence that a collective narrative-based approach to understanding polarization has advantages over previous approaches (that distinguish between polarization as issue driven or affective), as they are more descriptive of current polarization trends. We argue that polarization is most likely to occur when ideological conflicts are salient in society, as this fosters the formation of ideologically opposed camps which by their nature are prone to polarizing. Under this argument, there are two components that lead to polarization: when groups form around support for a particular narrative about social reality, and when this group exists in opposition to alternative viewpoints (e.g., Leavers and Remainers for Brexit). We term these ideologically opposed camps, based on support for a particular narrative, but we also suggest that these collective narratives diverge and can transform further as a consequence of intergroup and intragroup processes, thereby consolidating affective and issue driven polarization approaches and at the same time providing a potential pathway to radicalization. Applying this approach to current polarization trends, we examined evidence on contemporary conflicts and online polarization trends, finding that such a collective narrative approach can describe the increased polarization seen in modern politics. Further research into this theoretical paradigm may help develop interventions to reduce extreme polarization across fractured political fault lines, thereby improving democratic processes and social relations between people of varying opinions and beliefs.

## Author Contributions

A-MB developed the review plan and contributed to the review of research and writing of the manuscript. AB and KF contributed to the review of research and writing of the manuscript. All authors contributed to the article and approved the submitted version.

## Conflict of Interest

The authors declare that the research was conducted in the absence of any commercial or financial relationships that could be construed as a potential conflict of interest.

## Publisher’s Note

All claims expressed in this article are solely those of the authors and do not necessarily represent those of their affiliated organizations, or those of the publisher, the editors and the reviewers. Any product that may be evaluated in this article, or claim that may be made by its manufacturer, is not guaranteed or endorsed by the publisher.
